# Knockout of *SlMAPK3* enhances tolerance to heat stress involving ROS homeostasis in tomato plants

**DOI:** 10.1186/s12870-019-1939-z

**Published:** 2019-08-14

**Authors:** Wenqing Yu, Liu Wang, Ruirui Zhao, Jiping Sheng, Shujuan Zhang, Rui Li, Lin Shen

**Affiliations:** 1 College of Food Science and Nutritional Engineer Engineering, China Agricultural University, Beijing, 100083 China; 2 School of Agricultural Economics and Rural Development, Renmin University of China, Beijing, 100872 China

**Keywords:** Tomato plants, *SlMAPK3*, Heat tolerance, ROS, Antioxidant enzymes, *HSPs*, *HSFs*

## Abstract

**Background:**

High temperature is a major environmental stress that limits plant growth and agriculture productivity. Mitogen-activated protein kinases (MAPKs) are highly conserved serine and threonine protein kinases that participate in response to diverse environmental stresses in plants. A total of 16 putative *SlMAPK* genes are identified in tomato, and *SlMAPK3* is one of the most extensively studied *SlMAPKs*. However, the role of *SlMAPK3* in response to heat stress is not clearly understood in tomato plants. In this study, we performed functional analysis of *SlMAPK3* for its possible role in response to heat stress.

**Results:**

qRT-PCR analyses revealed that *SlMAPK3* relative expression was depressed by heat stress. Here, wild-type (WT) tomato plants and CRISPR/Cas9-mediated *slmapk3* mutant lines (L8 and L13) were used to investigate the function of *SlMAPK3* in response to heat stress. Compared with WT plants, *slmapk3* mutants exhibited less severe wilting and less membrane damage, showed lower reactive oxygen species (ROS) contents, and presented higher both activities and transcript levels of antioxidant enzymes, as well as elevated expressions of genes encoding heat stress transcription factors (*HSFs*) and heat shock proteins (*HSPs*).

**Conclusions:**

CRISPR/Cas9-mediated *slmapk3* mutants exhibited more tolerance to heat stress than WT plants, suggesting that *SlMAPK3* was a negative regulator of thermotolerance. Moreover, antioxidant enzymes and *HSPs*/*HSFs* genes expression were involved in *SlMAPK3*-mediated heat stress response in tomato plants.

**Electronic supplementary material:**

The online version of this article (10.1186/s12870-019-1939-z) contains supplementary material, which is available to authorized users.

## Background

High temperature, which causes heat stress, has become an increasingly serious agricultural problem in many regions of the world as a result of global warming [1]. Under heat stress, plant cells always show membrane damage, reactive oxygen species (ROS) overproduction and metabolic disturbance that further limit crop productivity and quality [2]. Tomato (*Solanum lycopersicum*) is a globally popular horticultural commodity with great economic importance, which also functions as a model plant species widely used in plant science, since it shows highly susceptible to diverse environmental stresses, such as drought, salinity, chilling, and heat.

In plants, mitogen activated protein kinase (MAPK) cascade have been reported to participate in signal transduction including plant development, hormone regulation, disease resistance, and stress responses [3]. There is increasing evidence that MAPK cascades play a vital role in mediating diverse cellular signaling network by transmitting extracellular stimuli to intracellular responses, which positively regulates gene expression and protein functions under various abiotic stresses, ultimately resulting in adaptive responses to environmental stresses [4]. The basic MAPK signaling modules are composed by an interlinked cascade of three consecutively acting protein kinases: MAPKK kinases (MAPKKKs), MAPK kinases (MAPKKs), and MAPKs, which are sequentially activated by phosphorylation. Previous studies reported that MAPK genes expression are significantly induced in response to heat treatment [5], and *AtMAPK6* in *Arabidopsis thaliana* [6], *ZmMAPK1* in maize [7], *MnMAPK1* in Mulberry [8], and *SlMAPK1* in tomato [9], have been shown to participate in heat stress response. However, there is a lack of report concerning the involvement of *SlMAPK3* in heat stress response.

In the tomato genome, 16 putative *SlMAPK* family genes have been identified, which can be clustered into four major groups (A–D) considering the similar exon-intron structures [4]. To date, *SlMAPK3*, a member in group A, is one of the most extensively studied *SlMAPKs* in tomato. It has been well documented that *SlMAPK3* plays an essential role in mediating a diversity of biotic and abiotic stress responses, including herbivorous insects [10], fungus [11], wounding [12], chilling [13], and drought [14]. Our previous studies revealed that knockout of *SlMAPK3* in tomato plant resulted in reduced drought tolerance and decreased disease resistance to *Botrytis cinerea* [11, 14]. However, the specific role of *SlMAPK3* in response to heat stress is not understood in tomato plants.

Oxidative damage on cell membranes has been implicated as a common event under abiotic stress that is assessed by the increase in both MDA content and ion leakage level [15]. Besides, oxidative damage can be ascribed to the overproduction of ROS, and both the formation and the scavenging of ROS are important for maintaining the steady state levels of ROS. The NADPH oxidase encoded by respiratory burst oxidase homolog (*RBOH*) genes is the major source of ROS in plants [16–18]. Previous studies documented that suppressed transcript level of *SlRBOH1* compromised BR-induced activation of *SlMAPK1/2* and *SlMAPK3*, and silencing of either *SlMAPK1* or *SlMAPK2* reduced *SlRBOH1* transcript level and H_2_O_2_ accumulation [16, 17]. Moreover, *SlMAPKs* have been reported to participate in the regulation of defense response against abiotic stress by scavenging excess ROS [9, 14, 19]. For example, knockout of *SlMAPK1* enhanced tolerance to heat stress by elevating antioxidant enzymes activities, which are crucial in ROS scavenging [9]. Our previous study showed that *slmapk3* mutants were sensitive to drought stress, with lower antioxidant enzymes activities and higher H_2_O_2_ content [14]. However, the relationship between ROS and *SlMAPK3*-mediated heat tolerance still remains unclear.

In plants, heat shock proteins (HSPs), including HSP100, HSP90, HSP70, HSP60, and small HSPs (smHSPs) [20], are generally considered as important molecular chaperones that contribute to maintain and/or restore protein homeostasis, which are crucial for plant survival under heat stress [21]. In addition, heat stress transcription factors (HSFs) are responsible for heat stress-induced gene expression [22]. Examples of this is that, under heat stress, HSFs can regulate *HSPs* genes expression by binding to heat stress elements (HSE: 5′-AGAAnnTTCT-3′) that are presented in the promoters of *HSPs*, ultimately inducing the responsiveness of downstream genes to heat stress [22]. It was previously reported that under heat stress, AtMAPK6 could phosphorylate HSFA2, and the phosphorylated-HSFA2 played an important role in response to heat stress [6]. In tobacco, a heat-activated MAP kinase (HAMK) functioned as a regulator in heat response, and heat induced expression of *HSF1/2* required the existence of MAPKK [23]. Thus, there might be correlations between HSPs/HSFs and MAPK-associated signaling pathways under heat stress.

In this study, relative expression of *SlMAPK3* was examined after different high temperature treatments in tomato plants, and CRISPR/Cas9-mediated *slmapk3* mutants (L8 and L13) were applied to investigate the role of *SlMAPK3* in response to heat stress. Our current results demonstrated that knockout of *SlMAPK3* could enhance heat tolerance, reduce ROS accumulation, and upregulate several *HSPs/HSFs* genes expressions in tomato plants, which implied that *SlMAPK3* acted as a negative regulator of defense response to heat stress.

## Results

### Analysis of expression patterns of *SlMAPK3* under different temperature conditions

**Fig. 1:**
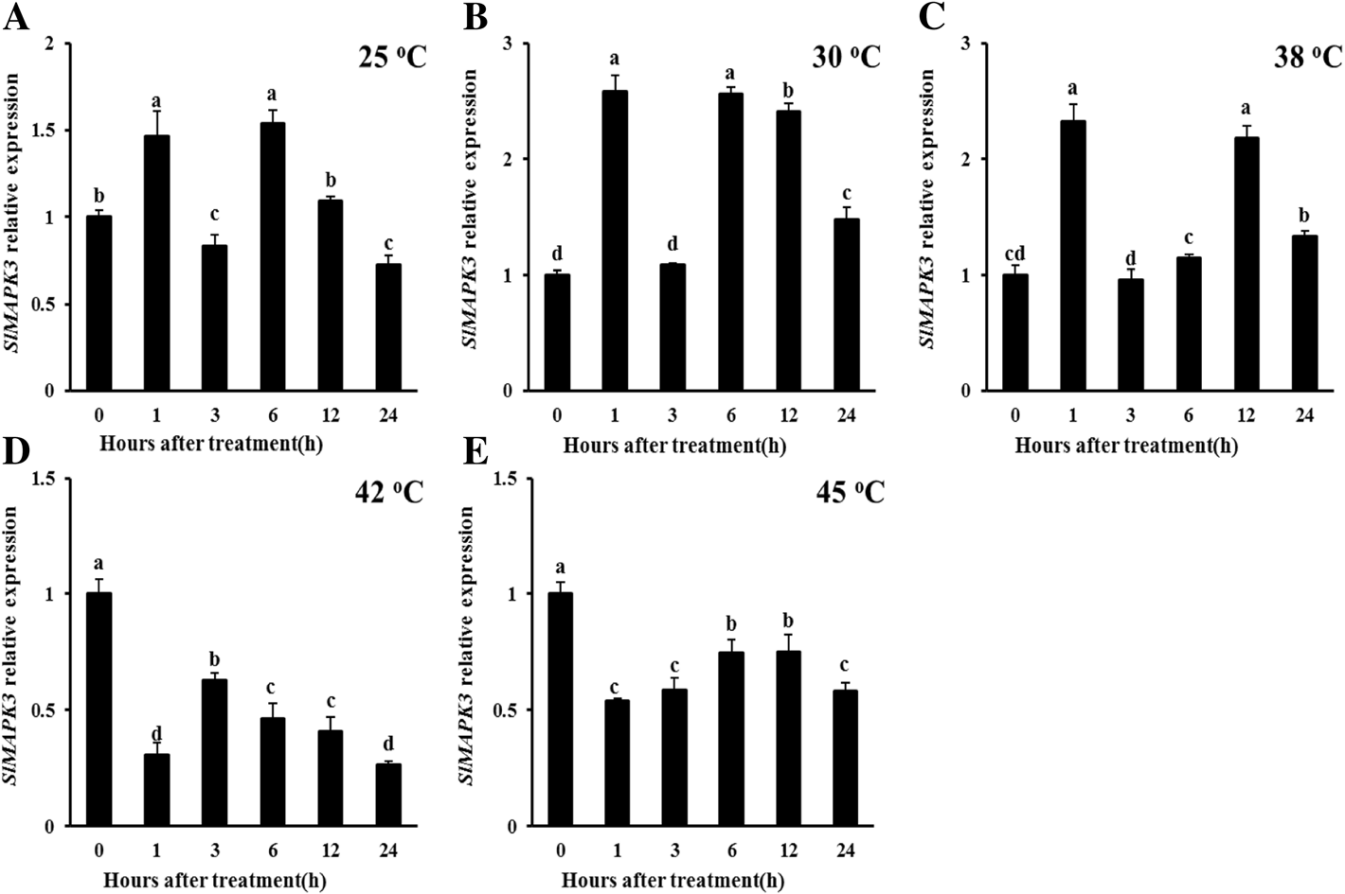
Expression analysis of *SlMAPK3* under different temperature conditions in WT tomato plants. (**a**) at 25 °C, (**b**) at 30 °C, (**c**) at 38 °C, (**d**) at 42 °C, (**e**) at 45 °C. Data are represented as mean ± SD of three biological replicates. Statistical differences at each time point of treatment are labeled with different letters according to Duncan’s multiple range test at *P* < 0.05

Transcript levels of *SlMAPK3* at different temperatures (25, 30, 38, 42, and 45 °C) were investigated by qRT-PCR (Fig. 1, *P* < 0.05). Our results showed that *SlMAPK3* relative expressions were increased at 25, 30, and 38 °C, which showed fluctuating changes (Fig. 1a, b and c, *P* < 0.05). On the contrary, when WT plants were exposed to higher temperature treatments (42 and 45 °C), the transcript levels of *SlMAPK3* significantly reduced after heat treatment (Fig. 1d and e, *P* < 0.05). These results indicated that *SlMAPK3* might participate in heat response.

### Phenotype of *slmapk3* mutants under heat stress

**Fig. 2:**
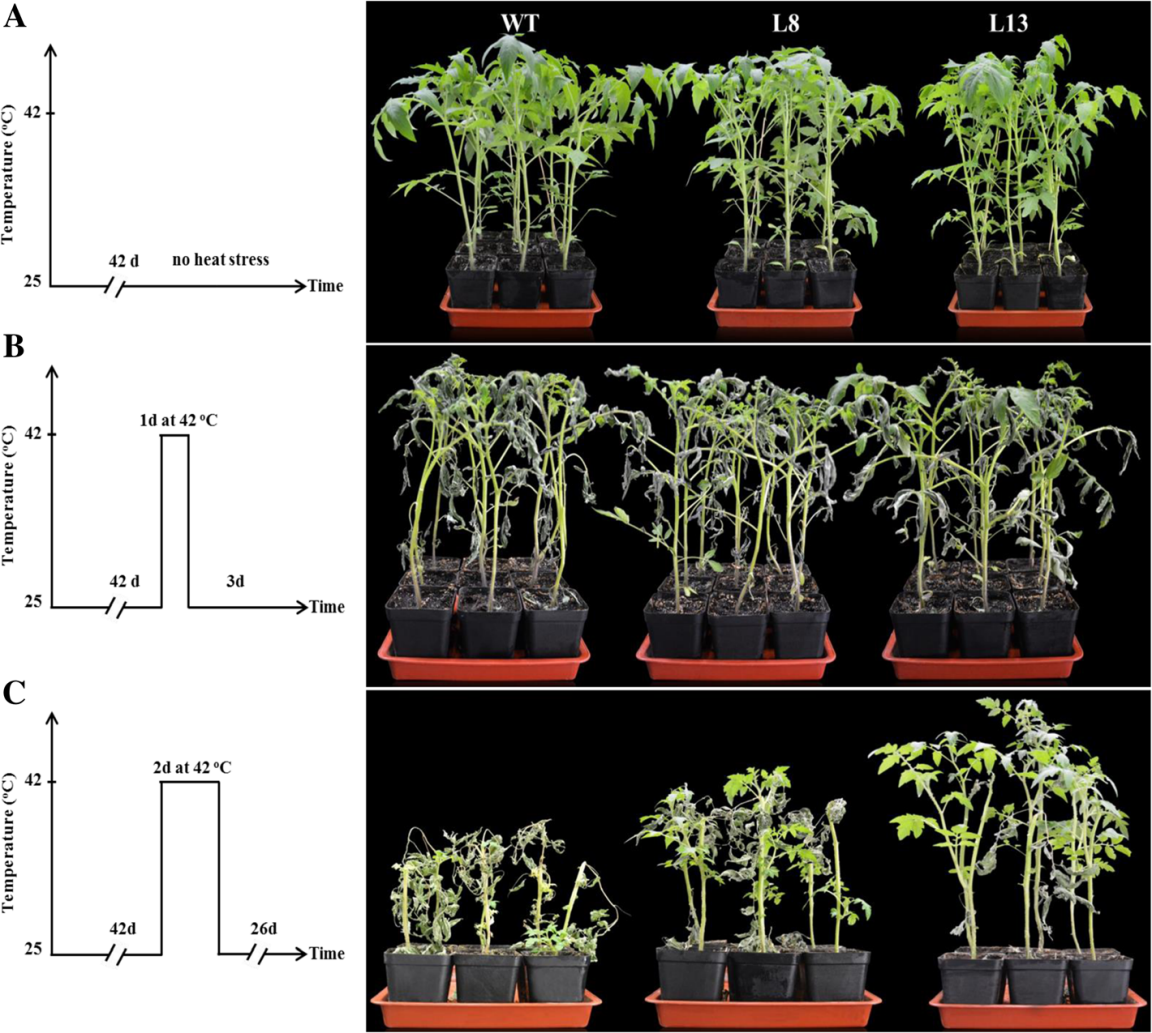
Phenotype of *slmapk3* mutants and WT plants under heat stress. **a** Six-week-old tomato plants of *slmapk3* mutants and WT under normal conditions. **b** Six-week-old tomato plants of *slmapk3* mutants and WT were subjected to 42 °C for 1 d then placed them to 25 °C for 3 d. **c** Six-week-old tomato plants of *slmapk3* mutants and WT were subjected to 42 °C for 2 d then placed them to 25 °C for 26 d

Before heat treatment, no significant differences between *slmapk3* mutants and WT plants could be observed (Fig. 2a). However, after 1 day’s exposure to 42 °C, visible symptoms of leaves wilting and stem bending were aggravated in WT plants, compared with *slmapk3* mutants which showed less severe wilting (Fig. 2b). A similar result could also be observed when heat stress was prolonged to 2 d, and the severest symptom was exhibited in WT plants (Fig. 2c). Meanwhile, the survival rate in *slmapk3* mutants were 3.17 (in L8) and 4.17 (in L8) times higher than that in WT plants (Additional file 1: Figure S1). These results indicated that knockout of *SlMAPK3* enhanced heat tolerance in tomato plants, suggesting that *SlMAPK3* played a negative role in response to heat stress.

### Effects of *slmapk3* mutants on cell membrane damage, MDA content, and ion leakage under heat stress

**Fig. 3:**
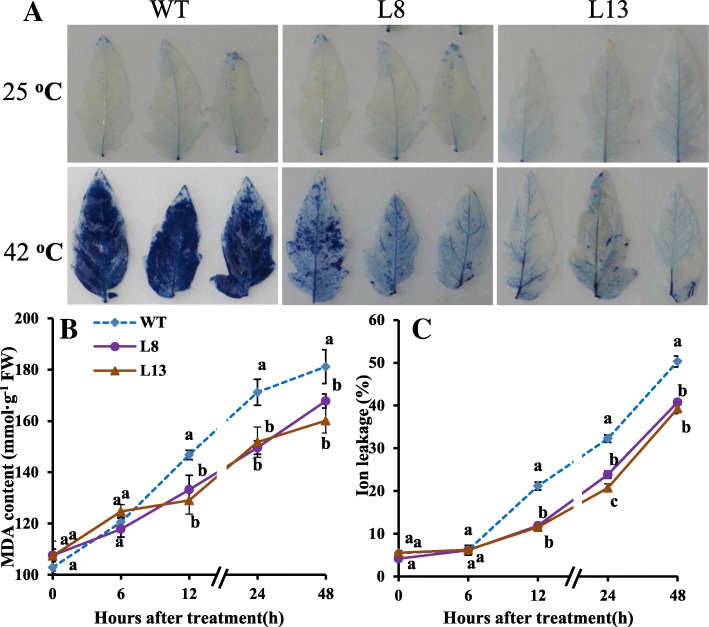
Effects of *slmapk3* mutants on cell membrane damage under heat stress. **a** Trypan blue staining, **b** MDA content, **c** ion leakage level. For (**a**), the top panel represents leaves grown at 25 °C, and the bottom panel represents leaves treated with 42 °C for 24 h. Data are represented as mean ± SD of three biological replicates. Statistical differences at each time point of treatment are labeled with different letters according to Duncan’s multiple range test at *P* < 0.05

Cell membrane damage was analyzed by trypan blue staining. Under normal conditions, there was no significant difference between WT and *slmapk3* mutants. However, after 42 °C treatment for 24 h, staining intensity in *slmapk3* mutants was lower than that in WT (Fig. 3a). Ion leakage and MDA content are physiological indices of cell membrane damage. Under normal conditions, MDA content and ion leakage were not significantly different between WT and *slmapk3* mutants (Fig. 3b and c, *P* < 0.05). In contrast, MDA content and ion leakage were significantly increased under heat stress, and MDA contents and ion leakage levels were remarkably higher in WT plants, in comparison with *slmapk3* mutants (Fig. 3b and c, *P* < 0.05).

### Effects of *slmapk3* mutants on ROS accumulation under heat stress

**Fig. 4:**
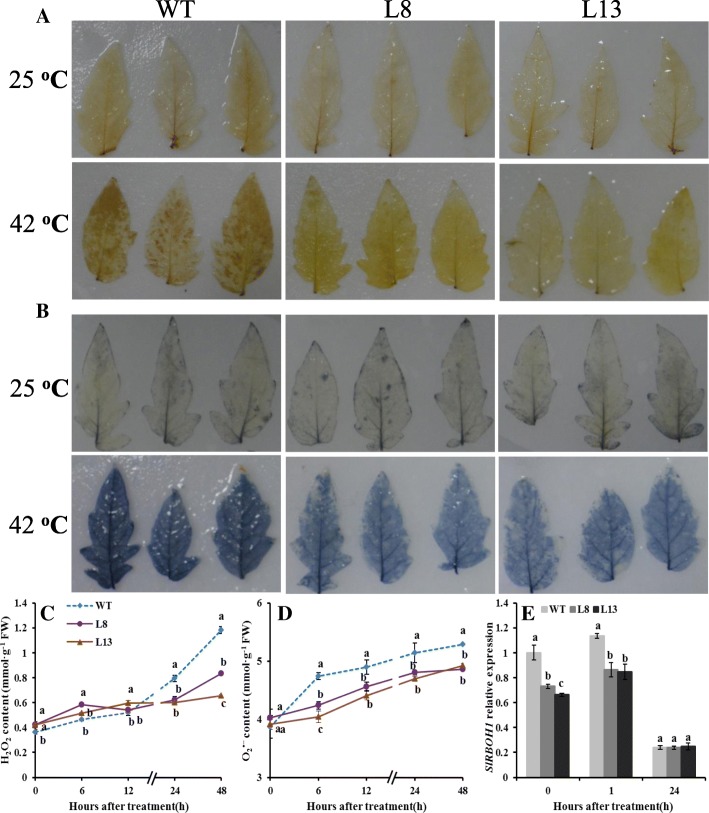
Effects of *slmapk3* mutants on ROS production under heat stress. **a** DAB staining, **b** NBT staining, **c** H_2_O_2_ content, **d** O_2_^•−^ content, and (**e**) *SlRBOH1* relative expression. For (**a**) and (**b**), the top panel represents leaves grown at 25 °C, and the bottom panel represents leaves treated with 42 °C for 24 h. Data are represented as mean ± SD of three biological replicates. Statistical differences at each time point of treatment are labeled with different letters according to Duncan’s multiple range test at *P* < 0.05

The accumulations of H_2_O_2_ and O_2_^•−^, which are two major components of ROS, were detected by DAB and NBT staining. After heat treatment for 24 h, DAB and NBT staining results showed that *slmapk3* mutants accumulated less H_2_O_2_ and O_2_^•−^ than WT (Fig. 4a and b). This was consistent with the results of quantitative analysis, in which H_2_O_2_ and O_2_^•−^ contents were 28.0, 7.1% (in L8), and 32.5, 9.4% (in L13) lower than those in WT plants at 24 h (Fig. 4c and d, *P* < 0.05).

Under normal conditions, *SlRBOH1* transcript levels in *slmapk3* mutants were significantly lower than that in WT plants (Fig. 4e, *P* < 0.05). After 1 h of heat stress, *SlRBOH1* transcript levels increased both in WT and in *slmapk3* mutants, but transcript levels in L8 and L13 were 23.9 and 25.5% lower than that in WT plants (Fig. 4e, *P* < 0.05). Moreover, after 24 h of heat stress, *SlRBOH1* transcript levels decreased dramatically, and no significant difference was observed between WT and *slmapk3* mutants (Fig. 4e, *P* > 0.05). Taken together, these results indicated that knockout of *SlMAPK3* reduced the overproduction of ROS under heat stress.

### Effects of *slmapk3* mutants on antioxidant enzymes under heat stress

**Fig. 5:**
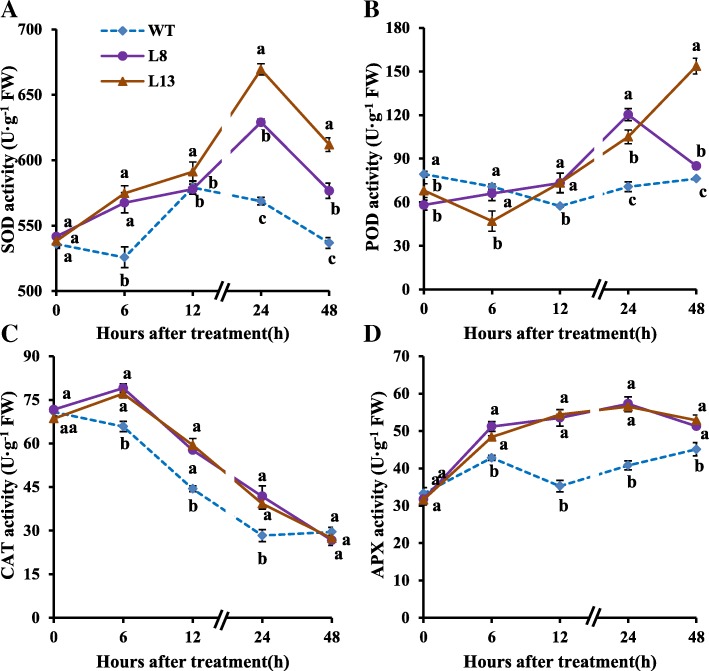
Effects of *slmapk3* mutants on antioxidant enzymes activities under heat stress. **a** SOD, **b** POD, **c** CAT, and **d** APX. Data are represented as mean ± SD of three biological replicates. Statistical differences at each time point of treatment are labeled with different letters according to Duncan’s multiple range test at *P* < 0.05

During the whole stress period, *slmapk3* mutants showed significant higher SOD activities than those in WT plants except the twelfth hour (Fig. 5a, *P* < 0.05). POD activities in WT plants reduced gradually from hours 6 to 12 and then increased afterward. POD activities in *slmapk3* mutants showed a fluctuating increase and were significantly higher than those in WT plants after 12 h heat treatment (Fig. 5b, *P* < 0.05). CAT activities reached a maximum of 79.0 U·g^−1^FW in L8 and 77.1 U·g^−1^FW in L13 on the sixth hour and then declined, but they were significantly higher than those in WT plants up to 24 h after heat treatment (Fig. 5c, *P* < 0.05). APX activities in *slmapk3* mutants increased gradually after heat treatment for 24 h, the average CAT activities in *slmapk3* mutants were 16.3, 53.1, 39.5 and 15.4% higher than those in WT plants at hours 6, 12, 24 and 48, respectively (Fig. 5d, *P* < 0.05).

**Fig. 6:**
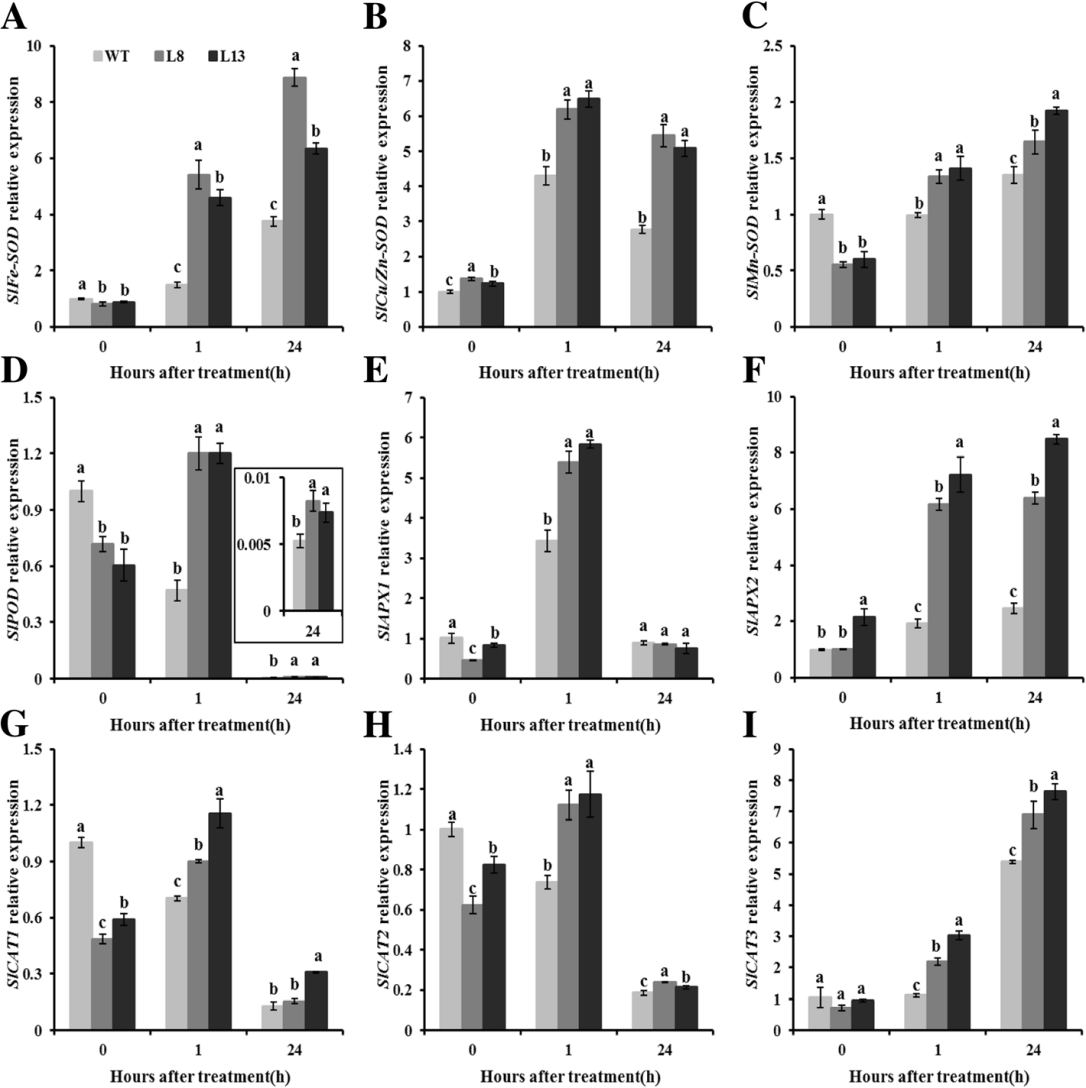
Effects of *slmapk3* mutants on the transcript levels of key antioxidant enzymes genes under heat stress. **a** *SlFe-SOD*, **b** *SlCu/Zn-SOD*, **c** *SlMn-SOD*, **d** *SlPOD*, **e** *SlAPX1*, **f** *SlAPX2*, **g** *SlCAT1*, **h** *SlCAT2*, and **i** *SlCAT3*. Data are represented as mean ± SD of three biological replicates. Statistical differences at each time point of treatment are labeled with different letters according to Duncan’s multiple range test at *P* < 0.05

Relative expressions of genes involved in encoding SOD, POD, CAT, and APX were assayed at the transcriptional level using qRT-PCR. In comparison with WT plants, transcript levels of *SlFe-SOD*, *SlMn-SOD*, *SlPOD*, *SlAPX1*, *SlCAT1*, *SlCAT2* and *SlCAT3* in *slmapk3* mutants were significantly lower under normal conditions (Fig. 6a, c, d, e, g, h and i, *P* < 0.05). After heat treatment, transcript levels of *SlFe-SOD*, *SlMn-SOD*, *SlAPX2*, and *SlCAT3* were upregulated, and the levels in *slmapk3* mutants were significantly higher than those in WT plants (Fig. 6a, c, f and i, *P* < 0.05). In addition, heat exposure enhanced the transcript levels of *SlCu/Zn-SOD*, *SlPOD*, *SlAPX1*, *SlCAT1* and *SlCAT2* at the time point of 1 h, and decreased those at 24 h in *slmapk3* mutants (Fig. 6b, d, e, g and h, *P* < 0.05). The changing patterns of *SlCu/Zn-SOD* and *SlAPX1* in WT plants were similar to the *slmapk3* mutants under heat stress, while the changing patterns of *SlPOD*, *SlCAT1* and *SlCAT2* were different from the *slmapk3* mutants, which decreased both at 1 h and at 24 h (Fig. 6b, d, e, g and h, *P* < 0.05). These results demonstrated that knockout of *SlMAPK3* increased both activities and transcript levels of *SOD*, *POD*, *CAT* and *APX* under heat stress.

### Effects of *slmapk3* mutants on gene expressions of *SlHSP70/90/100* and *SlHSFA1a/2/3* under heat stress

**Fig. 7:**
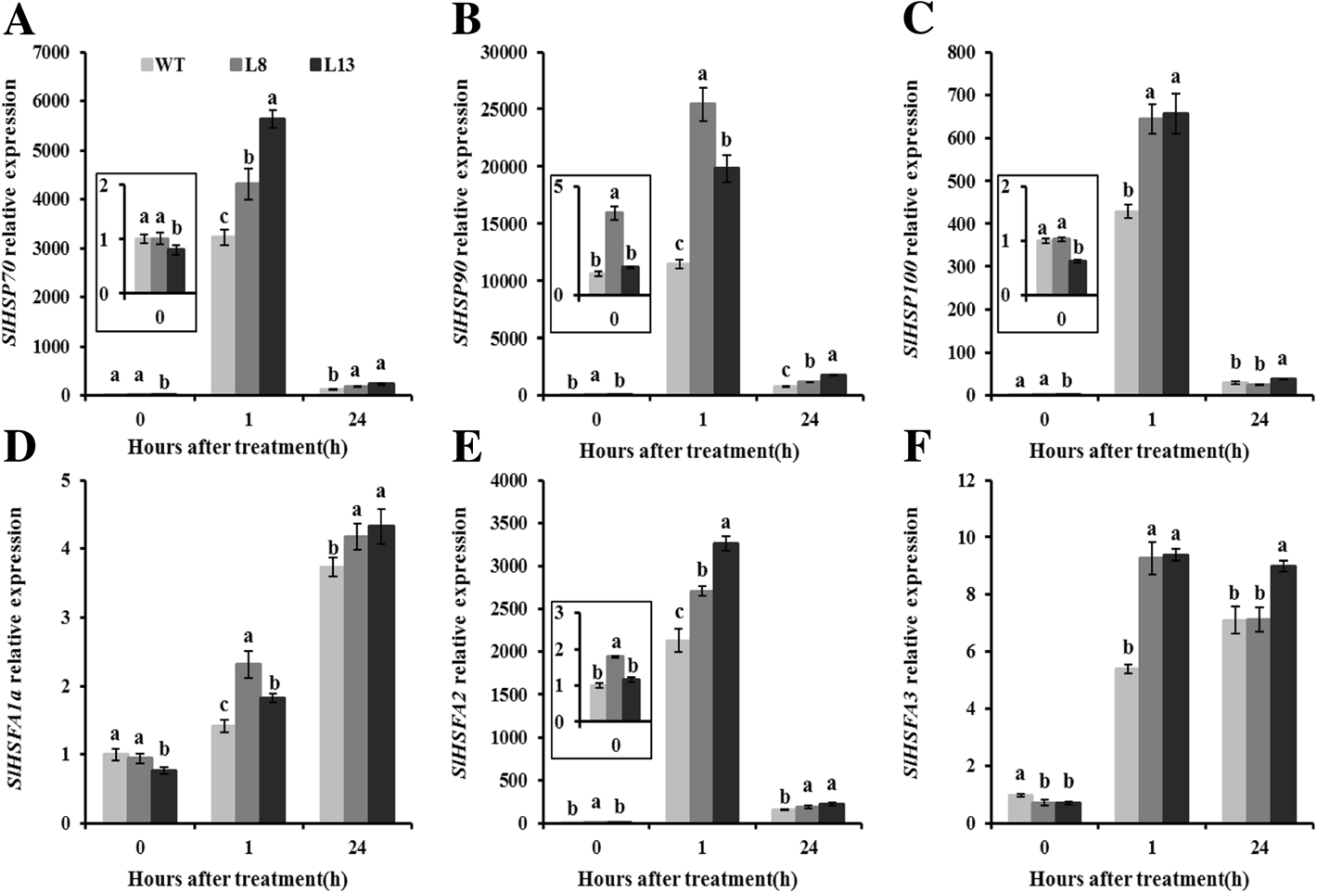
Effects of *slmapk3* mutants on the transcript levels of key *HSPs* and *HSFs* genes under heat stress. **a** *SlHSP70*, **b** *SlHSP90*, **c** *SlHSP100*, **d** *SlHSFA1a,* **e** *SlHSFA2,* and **f** *SlHSFA3*. Data are represented as mean ± SD of three biological replicates. Statistical differences at each time point of treatment are labeled with different letters according to Duncan’s multiple range test at *P* < 0.05

Apart from antioxidant system, another key adaptive mechanism developed by plant species when subjected to heat stress is the accumulation of heat shock response-related genes, including *HSPs* and *HSFs*. Therefore, relative expression of *SlHSP70*, *SlHSP90*, *SlHSP100* and *SlHSFA1a*, *SlHSFA2*, *SlHSFA3* were analyzed. After 1 h of heat stress, the transcript levels of *SlHSP70*, *SlHSP90*, *SlHSP100* and *SlHSFA2* were rapidly induced, and significant difference were observed between WT and *slmapk3* mutants. However, after 24 h of heat stress, transcript levels of these four genes decreased dramatically, but transcript levels in *slmapk3* mutants were still higher than those in WT plants (Fig. 7a, b, c and e, *P* < 0.05). The relative expressions of *SlHSFA1a* and *SlHSFA3* were significantly increased under heat stress, and *slmapk3* mutants showed higher levels of these two genes after 1 h and 24 h of heat stress compared with WT plants (Fig. 7d and f, *P* < 0.05).

### Correlation analysis of heat stress related physiological indexes

**Fig. 8:**
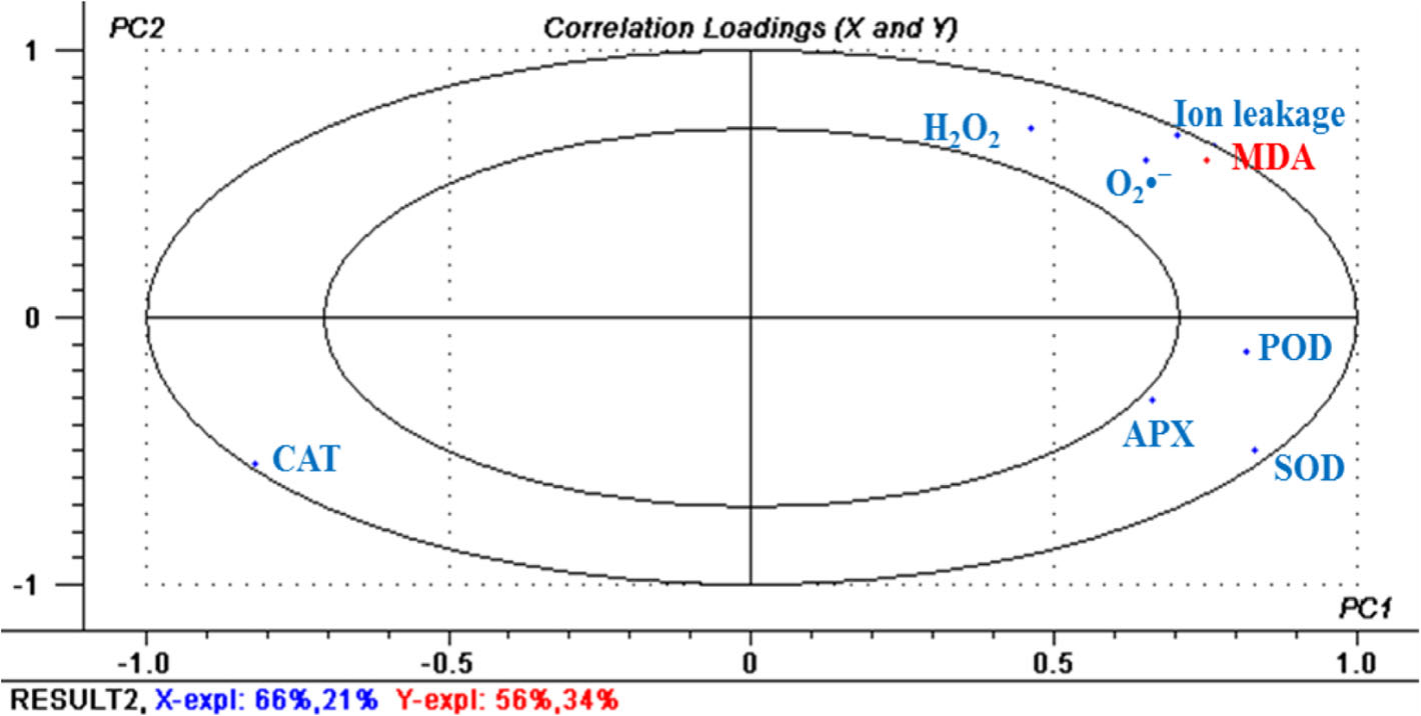
Partial least square regression analysis for physiological indexes of tomato plants under heat stress. The MDA content was used as Y-variables and other indexes as the X-variables

As shown in Table 1, positive correlations were found between MDA content and ion leakage, between MDA content and H_2_O_2_ content, between MDA content and O_2_^•−^ content, between ion leakage and H_2_O_2_ content, between ion leakage and O_2_^•−^ content, between H_2_O_2_ content and O_2_^•−^ content, between SOD activity and APX activity. In addition, negative correlations were found between MDA content and CAT activity, between ion leakage and CAT activity, between H_2_O_2_ content and CAT activity, between O_2_^•−^ content and CAT activity (*P* < 0.01). The significant correlations among MDA content, ion leakage, H_2_O_2_ and O_2_^•−^ contents could possibly be attributed to the fact that overproduction of ROS such as H_2_O_2_ and O_2_^•−^ caused oxidative stress, which resulted in lipid peroxidation and disruption of membrane integrity under heat stress [24]. Besides, the close relationship among CAT activity and MDA content, ion leakage, H_2_O_2_ and O_2_^•−^ contents (r > 0.72), indicated that CAT activity had significant negative correlations with the cell membrane damage and ROS accumulation under heat stress, which was consistent with a previous study that heat-induced decrease in CAT activity was strongly responsible for ROS detoxification in rice [25]. Furthermore, partial least square regression analysis (PLSR) results (Fig. 8) showed that MDA content was positively correlated with ion leakage, H_2_O_2_ and O_2_^•−^ content, and negatively correlated with CAT activity. Path analysis (PA) results (Table 2) provided further information that the influence of ion leakage on MDA content was achieved via direct impact, whereas the influence of H_2_O_2_ content, O_2_^•−^ content, POD activity and CAT activity on MDA content depended on other factors such as ion leakage.

**Table 1:** Pearson’s correlations among MDA content, ion leakage, H_2_O_2_ content, O_2_^•−^ content and antioxidant enzyme activities

	MDA content	Ion leakage	H_2_O_2_ content	O_2_^•−^ content	SOD activity	POD activity	CAT activity	APX activity
MDA content	1.000	0.947^b^	0.868^b^	0.921^b^	0.388	0.412	−0.936^b^	0.433
Ion leakage		1.000	0.883^b^	0.846^b^	0.245	0.492	−0.932^b^	0.310
H_2_O_2_ content			1.000	0.768^b^	0.071	0.188	−0.722^b^	0.339
O_2_^•−^ content				1.000	0.279	0.383	−0.873^b^	0.373
SOD activity					1.000	0.604^a^	−0.421	0.722^b^
POD activity						1.000	−0.575^a^	0.513
CAT activity							1.000	−0.326
APX activity								1.000

^a^Correlation is significant at the 0.05 level (2-tailed)

^b^Correlation is significant at the 0.01 level (2-tailed)

**Table 2:** Path coefficient analysis of characters on MDA content

Characters	Direct Effect	Indirect Effect								Correlation coefficient with MDA content
		Ion leakage	H_2_O_2_ content	O_2_^•−^ content	SOD activity	POD activity	CAT activity	APX activity	Total	
Ion leakage (X_1_)	0.550	–	0.051	0.226	0.046	−0.107	0.162	0.020	0.397	0.947
H_2_O_2_ content (X_2_)	0.057	0.486	–	0.206	0.013	−0.041	0.125	0.022	0.811	0.868
O_2_^•−^ content (X_3_)	0.268	0.465	0.044	–	0.052	−0.083	0.152	0.024	0.654	0.921
SOD activity (X_4_)	0.186	0.135	0.004	0.075	–	−0.131	0.073	0.046	0.202	0.388
POD activity (X_5_)	−0.217	0.271	0.011	0.103	0.112	–	0.100	0.033	0.629	0.412
CAT activity (X_6_)	−0.174	−0.512	−0.041	− 0.234	−0.078	0.124	–	−0.021	− 0.762	−0.936
APX activity (X_7_)	0.064	0.170	0.019	0.100	0.134	−0.111	0.057	–	0.370	0.433

Y = 0.642 + 0.906X_1_ + 7.024X_2_ + 14.760X_3_ + 0.119X_4_–0.200X_5_–0.224X_6_ + 0.173X_7_

R^2^ = 0.995

## Discussion

MAPKs are serine-threonine protein kinases that are highly conserved in eukaryotes [26]. In tomato, *SlMAPK3* has been studied extensively for its involvement in the responses to various stresses in plants, and silencing of *SlMAPK3* differently influence plant tolerance to multiple environmental stresses [11]. Our previous studies demonstrated that knockout of *SlMAPK3* in transgenic tomato plants resulted in reduced drought tolerance and decreased disease resistance to *Botrytis cinerea* in tomato plant, accompanied by lower antioxidant enzyme activity and higher H_2_O_2_ content [11, 14]. However, there is a lack of knowledge on the role and mechanisms of *SlMAPK3* in response to heat stress. In the present study, we showed that *SlMAPK3* relative expression was downregulated by high temperature treatments (42 °C, 45 °C) (Fig. 1d and e, *P* < 0.05), and knockout of *SlMAPK3* in CRISPR/Cas9-mediated mutagenesis showed more tolerance to heat stress than WT plants (Fig. 2), which suggested that *SlMAPK3* functioned as a negative regulator of heat response in tomato plants.

Generally, biological membranes are the first targets of diverse abiotic stresses, and loss of membranes integrity is a primary symptom of heat injury [27]. Previous studies documented that heat stress decreased membrane thermo-stability and increased the formation of membrane lipid peroxidation, as indicated by ion leakage and MDA content [28, 29]. Lower ion leakage level and MDA content could be observed in heat tolerant genotype, which have been successfully used as two important criteria for heat tolerant genotypes in tomato [30]. In this study, knockout of *SlMAPK3* alleviated heat stress-induced damage to the membrane system (Fig. 3a). Moreover, elevations in both ion leakage and MDA content were significantly lower in *slmapk3* mutants than in WT plants under heat stress (Fig. 3b and c, *P* < 0.05), which implied that knockout of *SlMAPK3* maintained the relative integrity of cell membrane and reduced cell membrane damage caused by heat stress.

Heat stress always leads to the overproduction of ROS. Excessive ROS generation in plant tissues can directly cause oxidative damage, ultimately impairing the normal function of cells [28]. It has been reported that ROS levels in heat-sensitive rice increased more profoundly than that in heat-tolerant rice under the same heat conditions, suggesting that there is a direct correlation between ROS accumulation and plant tolerance to heat stress [31]. The NADPH oxidase is the major source of ROS under various abiotic and biotic stresses*,* which is encoded by *RBOH* genes [18]. The tomato *SlRBOH1* has the highest transcript abundance within the *SlRBOH* family, which participates in the regulation of tolerance to heat stress [17, 32]. In our study, both H_2_O_2_ and O_2_^•−^ contents increased under heat stress, while the contents in *slmapk3* mutants were significantly lower than those in WT plants, and *SlRBOH1* transcript levels in *slmapk3* mutants were remarkably lower than that in WT plants after 1 h heat treatment (Fig. 4, *P* < 0.05). It’s indicated that knockout of *SlMAPK3* suppressed ROS overproduction, which contributed to alleviate cell membrane damage caused by high temperature (Table 1, Fig. 3, *P* < 0.05).

Antioxidant enzymes, including SOD, POD, CAT, and APX are crucial in ROS detoxification, which are thought to be a part of heat-stress adaptation, and their strengths are positively correlated with the acquisition of thermotolerance in plants [33, 34]. Besides, the activation of antioxidant enzymes played a crucial role in *MAPKs*-mediated stress responses including heat stress response*.* A good example of this is RNAi-*SlMAPK1* tomato plants, which showed higher heat tolerance than WT plants by increasing the antioxidant enzymes activities of SOD, POD, CAT, and APX [9]. In the present study, knockout of *SlMAPK3* significantly enhanced activities of these four antioxidant enzymes under heat stress by upregulating relative expression of their corresponding genes (Figs. 5 and 6, *P* < 0.05), which helped to scavenge ROS and alleviate oxidative damage (Figs. 3 and 4, *P* < 0.05). These results indicated that antioxidant enzymes were involved in heat stress response mediated by *SlMAPK3*.

Currently, the role of *SlMAPK3* in the regulation of heat-stress-related genes is still not entirely understood. HSFs and HSPs are known to play important roles in enhancing thermotolerance of plants. Larger HSPs, especially HSP70 and HSP90, were reported to act as molecular chaperone that participated in upregulation of several downstream genes associated with heat response in plants [35, 36]. Previous studies indicated that ClpB/Hsp100 proteins were critical in governing plant thermotolerance, the antisense lines which exhibited an extreme suppression of *SlClpB/Hsp100* gene expression were hypersensitive to heat stress [37]. These studies supported our present data that *slmapk3* mutants had higher transcript levels of *SlHSP70*, *SlHSP90* and *SlHSP100* than WT plants (Fig. 7a, b and c, *P* < 0.05), suggesting that *slmapk3* mutants were more heat-resistant than WT plants. Moreover, three HSFs, namely HsfA1, HsfA2 and HsfB1, are critical components involved in mediating responsiveness of different heat stress-induced genes in tomato [22]. Thermotolerance was remarkably enhanced in *SlHsfA1a*-overexpressing lines, whereas the suppression lines exhibited heat-sensitive phenotypes [38]. Overexpression of *AtHsfA2* showed enhanced tolerance to heat stress, indicating a correlation between *HSFA2* expression level and heat tolerance in *Arabidopsis thaliana* [39]. In addition, ectopic overexpression of *SlHsfA3* conferred increased thermotolerance in *Arabidopsis thaliana* [40]. In our study, relative expressions of *SlHSFA1a*, *SlHSFA2* and *SlHSFA3* were significantly higher in *slmapk3* mutants than in WT plants under heat stress (Fig. 7d, e and f, *P* < 0.05). These results indicated that the increase in *HSPs* and *HSFs* genes relative expression might be associated with *SlMAPK3*-mediated heat stress response in tomato plants.

## Conclusions

**Fig. 9:**
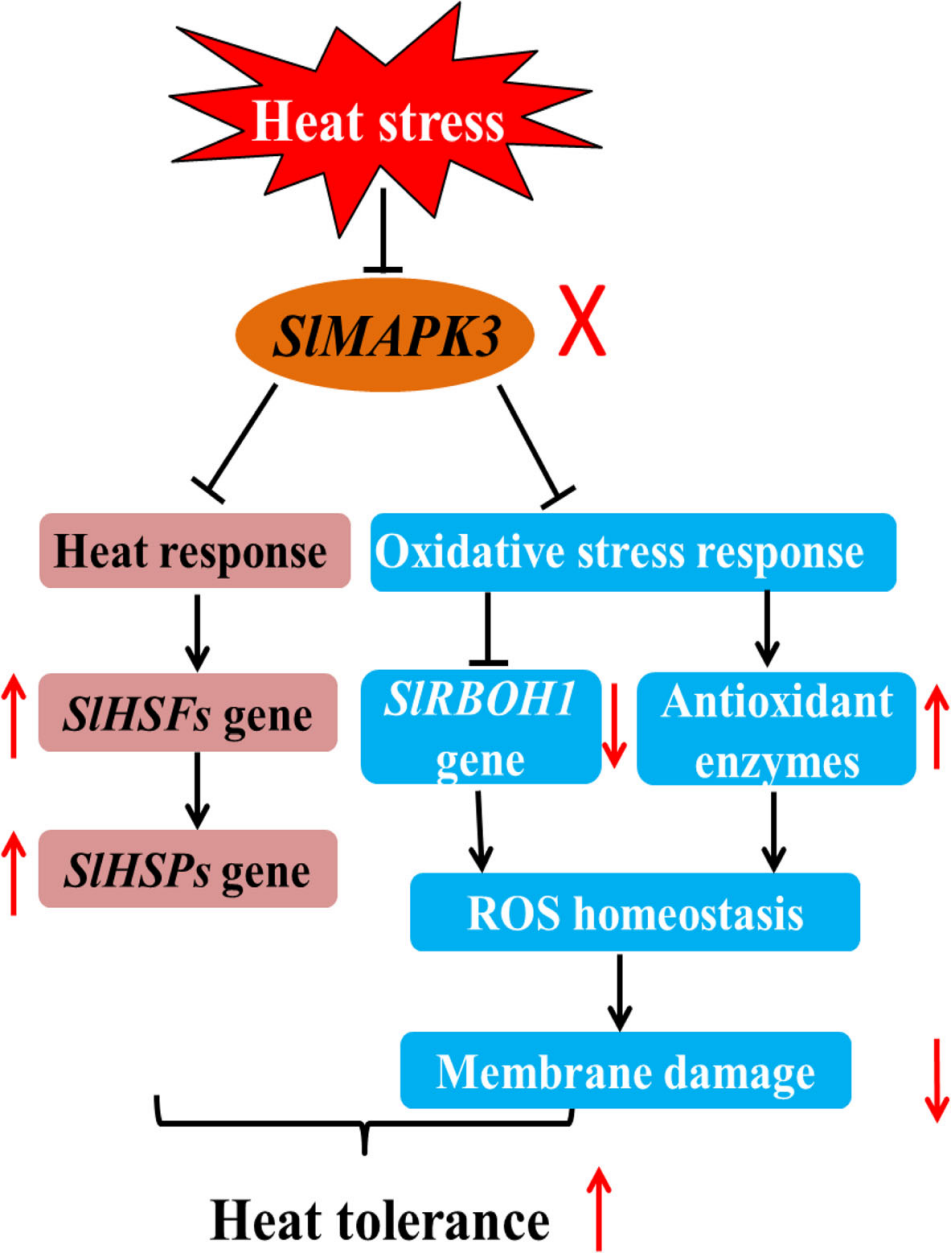
A proposed model of the role of *SlMAPK3* in tomato plants responses to heat stress involving ROS homeostasis. During heat stress, knockout of *SlMAPK3* suppressed heat-induced upregulation of *SlRBOH1* gene, and activated antioxidant enzymes, which alleviated ROS accumulation, in turn leading to a further mitigation in cell membrane damage in *slmapk3* mutants. The involvement of *SlHSFs/ SlHSPs* genes expressions are also suggested by solid lines. Consequently, prolonged heat stress tolerance is induced

In conclusion, our current study demonstrated that knockout of *SlMAPK3* enhanced heat tolerance in tomato plants. The decrease in MDA content and ion leakage implied that knockout of *SlMAPK3* prevented cell membrane from oxidative damage caused by heat stress. In addition, knockout of *SlMAPK3* reduced H_2_O_2_ and O_2_^•−^ contents, downregulated *SlRBOH1* relative expression, and increased both activities and transcript levels of *SOD*, *POD*, *APX* and *CAT*, suggesting that ROS production and scavenging were involved in *SlMAPK3*-mediated heat response (Fig. 9). Moreover, transcript levels of *SlHSP70*, *SlHSP90*, *SlHSP100* and *SlHSFA1a*, *SlHSFA2*, *SlHSFA3* were significantly higher in *slmapk3* mutants than those in WT plants, indicated that *SlHSFs* and *SlHSPs* genes might be involved in *SlMAPK3*-mediated heat response (Fig. 9). These results revealed that, the enhanced heat tolerance in *slmapk3* mutants could be associated with the suppression of ROS production and the activation of antioxidant enzymes, which led to lower ROS accumulation and alleviated oxidative damage under heat stress. Meanwhile, the transcript levels of *SlHSFs* and *SlHSPs* were also found to be modulated by *SlMAPK3* silencing. Taken together, this study suggests a possible regulatory mechanism involving *SlMAPK3*-mediated heat stress response, and provides insights into the role that MAPK cascade plays against abiotic stress in tomato plants. Further studies will pay more attention to the specific relationships between HSFs/HSPs and *SlMAPK3*-mediated heat response.

## Methods

### Plant materials and growth conditions

In this study, wild-type (WT) tomato plants (*Solanum lycopersicum* cv. Ailsa Craig) and T2 transgenic lines (L8 and L13) [14] were used. AC seeds were provided by Dr. Jim Giovannoni (Boyce Thompson Institute for Plant Research Ithaca, NY 14853, USA). All germinated tomato seeds were sown in plastic pots containing seedling substrate, soil, and vermiculite (2/1/1, by vol.), and grown in a greenhouse with a 16 h-light /8 h-dark photoperiod and 60–65% relative humidity, at a temperature of 25 °C.

For the heat stress tolerance assay, six-week-old plants were subjected to a 42/42 °C (day/night) illuminated chamber for two days. At each time point (0, 1, 3, 6, 12, 24 and 48 h) after treatment, five tomato plants were randomly selected and sampled from the same position. These samples were rapidly frozen in liquid nitrogen and stored at − 80 °C pending analysis. Three biological replicates were carried out in this experiment.

### Analysis of expression patterns of *SlMAPK3*

Six-week-old WT tomato plants were exposed to different high temperature treatments (25, 30, 38, 42 and 45 °C) in an illuminated chamber. After each treatment, functional leaves from the same position were sampled at 0, 1, 3, 6, 12 and 24 h, and then immediately frozen in liquid nitrogen and stored at − 80 °C for RNA extraction. The transcript level of *SlMAPK3* was measured by quantitative real-time PCR (qRT-PCR).

### qRT-PCR analysis

In this study, an *EasyPure* Plant RNA Kit (Beijing Transgen Biotech Co. Ltd., Beijing, China) was used to extract total RNA from 0.15 g frozen leaf sample. The total RNA was quantified by using a NanoDrop 2000 Photometer spectrophotometer (Thermo Scientific, Waltham, MA, USA), and 2 μg of RNA was reverse transcribed to synthesized cDNA by the aid of the *TransScript* One-Step gDNA Removal and cDNA Synthesis SuperMix Kit (Beijing Transgen Biotech Co. L td., Beijing, China).

The qRT-PCR was implemented using the *TransStart* Top Green qPCR SuperMix (Beijing Transgen Biotech Co.Ltd., Beijing, China), and the reaction mixture contains 5 μL of 2× SuperMix, 0.3 μL of both the forward and reverse specific primers (Additional file 2: Table S1), 1 μL of cDNA, and 3.4 μL of RNase-free water. The qRT-PCR was performed on a Bio-Rad CFX96 real-time PCR system (Bio-Rad, USA), and *β-Actin* was used as the reference gene. The expression levels of different genes were calculated using 2^-ΔΔCT^ method.

### Determination of MDA content and ion leakage

The lipid peroxidation and disruption of membrane integrity in cell membranes were estimated by measuring MDA content and ion leakage. MDA content was measured using the method as previously described by Ding et al. [41], and MDA content was expressed in mmol·g^− 1^ FW (fresh weight). Ion leakage was measured immediately from the leaf discs according to the method described by Zhao et al. [42], with some modifications.

### Determination of H_2_O_2_ content and O^2•−^ content

A H_2_O_2_ Detection Kit (A064, Jiancheng, Nanjing, China) and a superoxide radical anions (O_2_^•−^) Detection Kit (A052, Jiancheng, Nanjing, China) were used to assay the H_2_O_2_ and O_2_^•−^ contents, and both H_2_O_2_ and O_2_^•−^ contents were expressed as mmol·g^− 1^ FW.

### Histochemical detection of cell damage

Twenty-four hours after heat treatment, leaves from WT and *slmapk3* mutants were used for trypan blue staining analysis [43]. Detached leaves were soaked in 0.4% trypan blue solution at room temperature for 8 h. The photo was taken after decolorizing in boiling fixing liquid (lactic acid: glycerol: ethanol = 1:1:4).

### Histochemical detection of ROS

Nitroblue tetrazolium (NBT) and 3,3′-diaminobenzidine (DAB) were used to detect the accumulation of O_2_^•−^ and H_2_O_2_ as performed by Raina et al [44] Twenty-four hours after heat treatment, leaves from WT and *slmapk3* mutants were soaked in NBT (1 mg·mL^− 1^) or DAB (1 mg·mL^− 1^) solutions at room temperature for 8 h. The photo was taken after decolorizing in boiling 95% (v/v) ethanol.

### Determination of antioxidant enzyme activities

The activities of superoxide dismutase (SOD; EC 1.15.1.1), peroxidase (POD; EC 1.11.1.7), catalase (CAT; EC 1.11.1.6), and ascorbate peroxidase (APX; EC 1.11.1.11) were determined as previously described [45–48]. Frozen leaf sample (0.4 g, in powder form) was homogenized using an IKA Disperser in 5 mL of ice-cooled 100 mM potassium phosphate buffer (pH 7.0). The homogenate was centrifuged at 12000 *g* for 10 min at 4 °C, and then the supernatant was collected and used for antioxidant enzyme assays.

A SOD Detection Kit (A001, Jiancheng, Nanjing, China) was used to assay the SOD activity [45]. POD activity was assayed from the oxidation of guaiacol, and 1 unit of POD activity was defined as the 1 increase in absorbance at 470 nm per minute [46]. CAT activity was assayed from the consumption of H_2_O_2_, and 1 unit of CAT activity was defined as the 1 decrease in absorbance at 240 nm per minute [47]. APX activity was assayed by recording the absorbance of ascorbic acid at 290 nm, and 1 unit of CAT activity was defined as the 1 decrease in absorbance at 290 nm per minute [48]. All enzyme activities were calculated based on fresh weight, and were expressed as U·g^− 1^ FW.

### Statistical analysis

All data were obtained from three independent replicates, and the data were expressed as the mean ± standard deviation (SD). All statistical analyses were performed with SPSS 20.0 (IBM Corp., Armonk, NY). The data were analyzed by one-way analysis of variance (ANOVA). Mean separations were performed by Duncan’s multiple range tests. Differences with *P* < 0.05 were considered to be significant. Pearson’s correlation analysis was performed to determine the correlations among heat stress related physiological indexes. The PLSR model was constructed using the Unscrambler software (CAMO AS., Norway), and PA model was constructed using the SPSS 20.0 (IBM Corp., Armonk, NY).

## Additional files

Additional file 1: **Figure S1.** Survival rate of tomato plants described in Fig. 2c. (DOCX 4305 kb)

Additional file 2: **Table S1.** Sequences of specific primers used for qPCR analysis. (DOCX 31 kb)

## Data Availability

The datasets supporting the conclusions of this article are included within the manuscript and its additional files, and the raw data is available from the corresponding author on reasonable request.

## Abbreviations

APX: Ascorbate peroxidase
CAT: Catalase
CRISPR: Clustered regularly interspaced short palindromic repeats
DAB: DAB 3,3´diaminobenzidine
FW: Fresh weight
H_2_O_2_: Hydrogen peroxide
HAMK: Heat-activated MAP kinase
HSE: Heat stress elements
HSFs: Heat stress transcription factors
HSPs: Heat shock proteins
MAPKKKs: MAPK kinase kinases
MAPKKs: MAPK kinases
MAPKs: Mitogen-activated protein kinases
MDA: Malondialdehyde
NBT: Nitroblue tetrazolium
O_2_^•−^: superoxide radical anions
PA: Path analysis
PLSR: Partial least square regression analysis
POD: Peroxidase
qRT-PCR: quantitative real-time PCR
RBOH: Respiratory burst oxidase homolog
ROS: Reactive oxygen species
SD: Standard deviation
smHSPs: small HSPs
SOD: Superoxide dismutase
WT: Wild-type
