# Supplementary material for: Knockout of SlMAPK3 enhances tolerance to heat stress involving ROS homeostasis in tomato plants
Source: BMC Plant Biol. 2019 Aug 14;19:354. doi: 10.1186/s12870-019-1939-z (PMC6694692; doi:10.1186/s12870-019-1939-z)
Supplement: Supplementary file 1 — Figure S1. Survival rate of tomato plants described in Fig. 2c. (DOCX 4305 kb) [file 12870_2019_1939_MOESM1_ESM.docx]

**Supplementary Information**

**Knockout of *SlMAPK3* enhances tolerance to heat stress** **involving ROS homeostasis in tomato plants**

Wenqing Yu^1^, Liu Wang^1^, Ruirui Zhao^1^, Jiping Sheng^2^, Shujuan Zhang^1^, Rui Li^1^ and Lin Shen^1*^

^1^ College of Food Science and Nutritional Engineer Engineering, China Agricultural University, Beijing 100083, China

^2^ School of Agricultural Economics and Rural Development, Renmin University of China, Beijing 100872, China

* Corresponding Author

Lin Shen: Tel: +86-10-62737620; E-mail: shen5000@cau.edu.cn.

**Figure S1.** Survival rate of tomato plants described in Figure 2C.
