# Supplementary material for: Knockout of SlMAPK3 enhances tolerance to heat stress involving ROS homeostasis in tomato plants
Source: BMC Plant Biol. 2019 Aug 14;19:354. doi: 10.1186/s12870-019-1939-z (PMC6694692; doi:10.1186/s12870-019-1939-z)
Supplement: Supplementary file 2 — Table S1. Sequences of specific primers used for qPCR analysis. (DOCX 31 kb) [file 12870_2019_1939_MOESM2_ESM.docx]

**Supplementary Information**

**Knockout of *SlMAPK3* enhances tolerance to heat stress** **involving ROS homeostasis in tomato plants**

Wenqing Yu^1^, Liu Wang^1^, Ruirui Zhao^1^, Jiping Sheng^2^, Shujuan Zhang^1^, Rui Li^1^ and Lin Shen^1*^

^1^ College of Food Science and Nutritional Engineer Engineering, China Agricultural University, Beijing 100083, China

^2^ School of Agricultural Economics and Rural Development, Renmin University of China, Beijing 100872, China

* Corresponding Author

Lin Shen: Tel: +86-10-62737620; E-mail: shen5000@cau.edu.cn.

**Table S1**. Sequences of Specific Primers Used for qPCR Analysis

| name | accession number | forward primer (5’→3’) | reverse primer (5’→3’) |
| --- | --- | --- | --- |
| *SlMAPK3* | Solyc06g005170 | AGCATTAGCTCATCCCTACCTC | GCTCTTCTCCTATCCCTTGTTG |
| *SlRBOH1* | Solyc08g081690 | TGGGGATGACTACTTGAGCA | AAGCCTCGGAAAACACTCG |
| *SlFe-SOD* | Solyc06g048410.2.1 | GGGAAGTATCACAGGGCGTATG | GGCTCTCCTCCTCCGTTGG |
| *SlCu/Zn-SOD* | Solyc01g067740.2.1 | CCGACAAGCAGATTCCTCTC | TCATGTCCTCCCTTTCCAAG |
| *SlMn-SOD* | Solyc06g049080.2.1 | TTCTCTTGGCTGGGCTATTG | AGCACCTTCTGCGTTCATCT |
| *SlPOD* | Solyc01g006300.2 | CACATACATTTGGAAGGGC TTTATTGTTGGATCAGGGC | TTTATTGTTGGATCAGGGC |
| *SlCAT1* | Solyc12g094620.1.1 | GGTGGATTATTTGCCCTCG | ACCTCTCCCCTGCCTGTTT |
| *SlCAT2* | Solyc02g082760.2.1 | AACAACTTCCCCGTCTTCTTC | TTAGGATTTGGCTTCAGAGCA |
| *SlCAT3* | Solyc04g082460.2.1 | CCCTATTCCTCCTCGTGTCTT | TGTAATGTTCTCCTGGCTGCT |
| *SlAPX1* | Solyc06g005160.2.1 | CTGGTGTTGTTGCTGTTGAAG | GCTCTGGCTTGTCCTCTCTG |
| *SlAPX2* | Solyc06g005150.2.1 | GGCTGGTGTTGTTGCTGTTG | TCAGGCAAGCGACCTTCAAC |
| *SlHSP70* | Solyc11g020040.1 | CAAGCTGAAAGAGCTCAAGG | CTGTCCCAGCTGCATTACTT |
| *SlHSP90* | Solyc06g036290.2 | GAAGAAAAAGGAAGAGAAAAAA | CATACTCACCTGTCACTAAGCA |
| *SlHSP100* | Solyc02g088610.2 | GGACTGGGATTCCTGTTTCTAAG | GGATTGCCTCTGCTACTGCTCT |
| *SlHSFA1a* | Solyc08g005170 | AAATGATGTCGTTCCTGGC | ATCCTTCGTTTCTTGCTGC |
| *SlHSFA2* | Solyc08g062960 | TAGTGAAAGTGAAGGTCGAAGA | ATGAGAGTCCCAAACAATAAAG |
| *SlHSFA3* | Solyc09g009100 | ACTCCCTTTTGCTGACCC | CACACCCAATCCTCCCTC |
| *β-Actin* | Solyc03g078400.2 | CAGCAGATGTGGATCTCAAA | CTGTGGACAATGGAAGGAC |
